# BK channel activity in skin fibroblasts from patients with neurological disorder

**DOI:** 10.1080/19336950.2025.2542811

**Published:** 2025-08-10

**Authors:** Ria L. Dinsdale, Thomas R. Middendorf, Deborah Disilvestre, David Adams, William Gahl, Ellen F. Macnamara, Lynne Wolfe, Camilo Toro, Cynthia J. Tifft, Andrea L. Meredith

**Affiliations:** a Department of Physiology, University of Maryland School of Medicine, Baltimore, MD, USA; b Department of Neuroscience and Center for Learning and Memory, University of Texas at Austin, Austin, TX, USA; c NIH Undiagnosed Diseases Program, National Human Genome Research Institute, National Institutes of Health, Bethesda, MD, USA

**Keywords:** BK channel, K_Ca_1.1, calcium-activated potassium channel, *KCNMA1*, slowpoke, channelopathy

## Abstract

Seventy-five unique variants in the *KCNMA1* gene have been identified from individuals with neurological disorders. However, variant pathogenicity and evidence for disease causality are lacking in most cases. In this study, the *KCNMA1* variants N999S and E656A (rs886039469 and rs149000684, respectively) were investigated from two individuals presenting with neurological disorders. N999S was previously shown to produce strong gain-of-function (GOF) changes in homomeric BK channel properties *in vitro* and is found as a heterozygous allele associated with epilepsy and paroxysmal dyskinesia in humans. Although its pathogenicity has been demonstrated in heterozygous animal models, the GOF classification for N999S has not been validated in a heterozygous patient-derived tissue. Conversely, the GOF pathogenicity for E656A is based solely on homomeric channels expressed in vitro and is inconclusive. For either variant, the properties of single heterozygous channels and allele expression is unknown. In this study, we profiled the wild-type and mutant *KCNMA1* transcripts from primary human skin fibroblasts of heterozygous patients and unaffected controls and performed patch-clamp electrophysiology to characterize endogenous BK channel current properties. GOF gating was observed in single BK channel recordings from both channel types. Fibroblasts from the individual harboring the E656A variant showed decreases in the number of BK channels detected and E656A-containing transcripts compared to controls. These results show that single BK channels can be reliably detected in primary fibroblasts obtained from human skin biopsies, suggesting their utility for establishing variant pathogenicity, and reveal the BK channel expression and functional changes associated with two heterozygous patient genotypes.

## Introduction

*KCNMA1* (Potassium Calcium-Activated Channel Subfamily M Alpha 1) encodes the pore-forming α subunit of the large conductance voltage- and calcium-activated potassium (BK, big potassium) channel. BK channels are composed of a tetramer of *KCNMA1* α subunits [1]. *KCNMA1*-linked channelopathy, a rare disorder characterized by mutations in the *KCNMA1* gene, presents as neurological and neuromuscular dysfunction – specifically epilepsy, paroxysmal dyskinesia, and neurodevelopmental deficiencies [2]. Over 75 patient-associated *KCNMA1* variants have been identified [2]. A subset of *KCNMA1* variants have loss- (LOF) or gain-of-function (GOF) effects established from *in vitro* studies of homotetrameric channels (summarized in [2]). However, most individuals carry *de novo*, and therefore heterozygous, *KCNMA1* variants [2], creating the potential for heterotetrameric BK channels containing both mutant and wildtype subunits within patient tissues. The functional designations for BK channels from heterozygous patients have not been studied in most cases. Here, we focus on two patient-associated *KCNMA1* variants, N999S and E656A, which were selected based on the availability of patient-derived cells (primary skin fibroblasts) collected from two individuals enrolled in the NIH Undiagnosed Diseases Program at the NIH Clinical Center [3,4].

**Figure 1.**
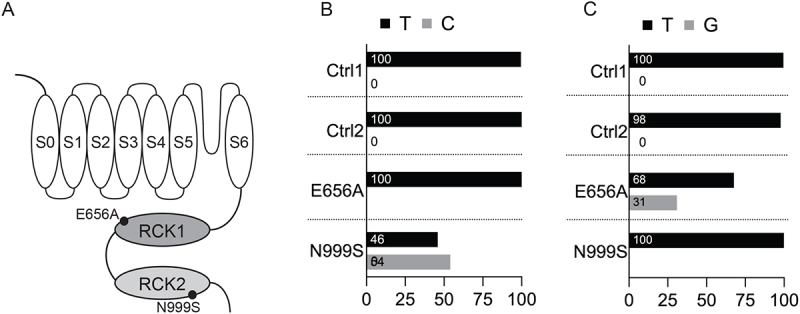
BK channel expression in skin fibroblasts. (A) BK channel linear sequence graphic showing the residues mutated in *KCNMA1*-N999S and *KCNMA1*-E656A patient fibroblasts. S, membrane-spanning region; RCK, regulator of potassium conductance domain. (B) RNA-sequencing analysis for the N999S variant showing the percentage of base counts for each allele. Number of reads were: 1142 (GM00038H or Ctrl-1); 337 (GM01652F or Ctrl-2); 237 (*KCNMA1*-E656A); 789 (*KCNMA1*-N999S). (C) RNA-sequencing variant analysis for the E656A variant showing the percentage of base counts for each allele. Number of reads: 1062 (Ctrl-1); 407 (Ctrl-2); 272 (E656A); 872 (N999S). In B and C, wild-type bases are shown in black, and mutant bases are shown in gray. The percentage of mutant or wild-type bases is shown inside each bar.

*KCNMA1*-N999S (also called N995S and N1053S in alternate reference transcripts) causes epilepsy and paroxysmal dyskinesia and is the most commonly identified pathogenic *de novo* variant in the *KCNMA1* gene [5–14]. N999 is located in the intracellular gating ring, near the interface between the RCK1 and RCK2 (regulator of conduction of K^+^) domains [15] (Figure 1(A)). Homomeric BK^N999S^ channels show increased BK channel current compared to wildtype (BK^WT^) channels, classifying as GOF [5,12,16].

In macroscopic recordings from heterologous expression systems, homomeric BK^N999S^ channel currents show a shift in the voltage dependence of activation to more hyperpolarizing membrane potentials, more rapid activation kinetics, and slower deactivation kinetics compared to WT channels [5,6,12,15,16]. In single-channel recordings, homomeric BK^N999S^ channels show a shift in the open probability versus voltage (Po-V) curve to more hyperpolarizing membrane potentials and a significantly prolonged channel open dwell time, in the absence of changes in the single-channel conductance or calcium-dependent activation [12]. Together, the hyperpolarized voltage-dependence and kinetic changes lead to a substantial increase in BK^N999S^ channel activity, even without intracellular calcium. Heterozygous BK^N999S/+^ transgenic mice exhibit GOF BK channel currents [5], and co-expression of WT and mutant transcripts also produces GOF channel properties in HEK cells [6]. However, the extent to which GOF effects are produced in human BK channels obtained from patient cells has not yet been investigated.

A second *KCNMA1* variant associated with epilepsy is E656A [12]. Only two individuals with neurological diagnoses have been reported to harbor this variant, and the pathogenicity of this mutation has not been fully established. E656 is located in the intracellular gating ring, distal to N999 within the RCK1 domain [15] (Figure 1(A)). Initial functional studies performed in heterologous cells under a symmetrical K^+^ gradient saw no effect of the E656A mutation on macroscopic BK channel currents [12]. However, a later study using a different BK channel isoform observed GOF channel behavior through a hyperpolarizing shift in V_1/2_ when compared to BK^WT^ under a physiological K^+^ gradient [15]. The magnitude of the hyperpolarizing shift in V_1/2_ produced by E656A was 2.5 times smaller than that produced by N999S (−17 mV and −44 mV, respectively) [15]. Neither study reported a difference in E656A channel kinetics compared to WT channels. Whether the discrepancies between these studies reflect contextual differences in splice variants and/or other factors has not yet been resolved. Furthermore, E656A has not been studied in the heterozygous conformation in any system.

The accuracy of *KCNMA1* variant assessments in homomeric BK channels expressed in heterologous systems in channelopathy disease is not known. In transgenic mice engineered to carry the *KCNMA1* GOF mutation D434G, differences were observed between homozygous and heterozygous mutant genotypes in neuronal firing patterns, seizure thresholds, and dyskinesia phenotypes [5,17]. Because most individuals harboring *KCNMA1* variants are heterozygous [2], a few studies have attempted to address the functional classifications from co-expressed mutant and wild-type channels. Macroscopic currents from HEK cells co-expressing BK^WT^ and BK^N999S^ channel plasmids showed intermediate behavior between WT and homozygous N999S channels [6]. In other experiments with equal transcript ratios of WT and the patient-associated variant *KCNMA1*^G375R^ expressed in oocytes to mimic heterozygosity, five different channel stoichiometries were observed [18]. All except those likely to be homomeric WT channels showed GOF behavior, implying genetic semi-dominance from an equal ratio of WT and mutant transcripts. However, the relative expression of mutant and WT alleles has not been established in heterozygous individuals presenting with neurological disorder. These examples demonstrate the importance of understanding the expression and properties of BK channels formed by WT and mutant alleles from patient tissues, toward more accurate genotype-phenotype correlation.

In this study, we evaluated *KCNMA1* transcript levels and BK channel activity from primary human skin fibroblast cells obtained from two heterozygous patients harboring *KCNMA1* N999S and E656A mutations. RNA sequencing revealed differences in allele expression in *KCNMA1*^E656A/+^, but not *KCNMA1*^N999S/+^, skin fibroblasts. Cell-attached patch clamp recordings of single BK channels showed that both mutations produced GOF effects, but to differing degrees. These data corroborate GOF functional classifications for heterozygous N999S and E656A in patient-derived primary tissue and suggest how differences in expression may further influence mutant channel phenotypes.

## Materials and methods

### Patient genotyping and skin fibroblasts

This study was approved by the National Institutes of Health (NIH) Institutional Review Board (IRB) on Human Subject Research under IRB 15-HG-0130 (Clinical and Genetic Evaluation of Patients with Undiagnosed Disorders Through the Undiagnosed Diseases Network) and 76-HG-0238 (Diagnosis and Treatment of Patients with Inborn Errors of Metabolism or Other Genetic Disorders), and written informed consent was obtained from the study subjects or their parents. The study abides by the Declaration of Helsinki principles. Case 1 is a biracial (African American and white) female who had a full onsite clinical workup and skin punch biopsy performed at the NIH Clinical Center at age 3. Whole-genome sequencing was performed at HudsonAlpha Clinical Services Lab (Huntsville, AL). Primary fibroblasts from this case are called “*KCNMA1*^N999S/+^” in this study. Case 2 is a Hispanic female adopted from Columbia and raised in USA. She was seen at the NIH CC at age 39 for a skin punch biopsy and for entry of her retrospective medical history into the NIH clinical record. Prior genetic testing had been performed by Athena Diagnostics with an epilepsy panel (141 genes) at age 23. PCR sequencing for the EXPH5 gene was performed at Columbia University Laboratory of Personalized Genomic Medicine at age 25. Additional retrospective self-reported medical history was obtained under University of Maryland Baltimore IRB non-human subject research designations (NHSR) HP-00083221 and HP-00086440. Primary fibroblasts from this case are called “*KCNMA1*^E656A/+^.”

De-identified normal control skin fibroblasts were obtained from the Apparently Healthy Collection of the NIGMS Human Genetic Cell Repository at the Coriell Institute (Camden, New Jersey, USA) under University of Maryland Baltimore NHSR designation HP-00107844. Control 1 (Ctrl-1; catalog #GM00038) was obtained from an unaffected 9-year-old female (black/African American), and Control 2 (Ctrl-2; catalog #GM01652) was obtained from an unaffected 11-year-old female (white).

### Cell culture

Primary human patient fibroblast lines were established from punch skin biopsy samples and maintained in DMEM media (Cat. #11995–065, Gibco, Life Technologies Corp., Grand Island, NY, USA) supplemented with: 10% fetal bovine serum (Cat. #100–106, GeminiBio, West Sacramento, California, USA), 1% penicillin/streptomycin (Cat. #400–109, GeminiBio, West Sacramento, California, USA) and 1% L-glutamine (Cat. #25–005-Cl, Mediatech Inc., Manassas, VA, USA) in a humidified incubator at 37°C with 5% CO_2_. Fibroblasts were passaged twice weekly and recorded at passages 4–12 by splitting onto glass coverslips precoated with poly-L-lysine (Sigma; P4832). Outward K^+^ currents were recorded 1–3 days later.

### RNA sequencing (RNAseq)

Fibroblasts at approximately 90% confluency were harvested by trypsinization of the monolayers, pelleted, and rinsed in DBPS. Total cell count and passage numbers (P) were: GM00038H (Ctrl-1), 6.0 × 10^6^, P16; GM01652 (Ctrl-2), 3.4 × 10^6^, P14; E656A, 7.5 × 10^6^, P8; and N999S, 3.4 × 10^6^, P9. Cells were flash-frozen in liquid nitrogen and stored at −80°C. Total RNA was extracted with the Qiagen RNeasy Mini kit (Qiagen, Venlo, the Netherlands) according to manufacturer’s protocol for cultured animal cells. RNA was quantified on an Agilent 2100 Bioanalyzer (Agilent Technologies, Palo Alto, CA, USA). Samples with an RNA integrity score (RIN) higher than 9 qualified for further processing. 800 ng of total RNA from each sample was then Poly-A enriched to minimize ribosomal RNA background, using the NEBNext Poly(A) mRNA Magnetic Isolation Module (New England Biolabs, Ipswitch, MA).

Strand-specific, indexed libraries were made using the NEBNext® Ultra™ II Directional RNA Library Prep Kit for Illumina® (New England Biolabs, Ipswich, MA) according to the manufacturer’s protocols, except that 3 µl of a 1:30 dilution of adapter was used. Size selection was performed with SPRI-select beads (Beckman Coulter Genomics, Danvers, MA). The final amplification and glycosylase digestion were performed concomitantly.

RNA sequencing was carried out on an Illumina NovaSeq 6000 paired-end 2 × 150bp run by Maryland Genomics, Institute for Genome Sciences, UMSOM. Following sequencing, raw data from the sequencer was processed using Illumina and Maryland Genomics-developed pipelines for sequence assessment and quality control. After base-calling was performed by the Illumina pipelines, our quality control pipeline assessed the base call quality and truncated reads where the median Phred-like quality score fell below Q20. On average, 170 M read pairs (340 M reads) per sample were generated and retained following sequencing and data processing.

The resulting paired-end reads were mapped to the reference human genome GRCh38.108 (Ensemble annotation) using HiSat2 v2.1.0, and default mismatch parameters resulting in 97% properly paired read mapping for each sample. The resulting bam file was visualized using IGV-2.7.2 to confirm the variant calls within the loci of interest. The pileup of reads at the position of interest was calculated using samtools (v 1.11) mpileup.

### Electrophysiological recordings and channel analysis

Single- and multi-channel BK currents were recorded in cell-attached configuration at room temperature with a Multiclamp 700B and Digitizer 1550B. The external (bath) solution contained (mM): 140 K-Methanosulphanate, 2 KCl, 5 HEDTA and 20 HEPES. Electrodes (4–8 MΩ) were filled with a pipette solution containing (mM): 140 K-Methanosulphanate, 2 KCl, 2 MgCl_2_ and 20 HEPES. Currents were elicited at potentials from −40 mV to 160 mV for 15 s in 20 mV increments, returning to a holding potential of 0 mV between each step. In some experiments, 100 nM paxilline (AlomoneLabs, Jerusalem, Israel; *p*-450) was applied after baseline recordings to confirm the identity of BK K^+^ currents.

Recordings were digitized at 50 kHz and filtered online at 6 kHz. Representative traces were filtered offline at 1 kHz. NPo and Po were determined over 15 seconds of recording, using 50% threshold analysis [19] in Clampfit (v11, Molecular Devices, USA). Where the number of channels per patch was >1, the average Po was calculated in Clampfit as NPo/N. Throughout the text, the reported voltages are membrane potentials.

### Histograms and mean dwell time distributions

Current amplitude peaks were plotted from an all-points histogram for each cell, and the current level between the closed and open histogram peaks was set as the threshold for event detection for idealization of the current records. Open and closed dwell-time histograms were generated using bins of constant width on a logarithmic x-axis. This coordinate transform is useful because the position of the peak in each histogram component is equal to the corresponding time constant [20]. Open-time histograms were fitted in Igor Pro v9 Build 37,840 (WaveMetrics, Inc., Lake Oswego, Oregon) using a single-component log-normal distribution function to estimate the open-time constant (τ_open_). Closed-time histograms were fitted in Igor Pro using a two-component (double) log-normal distribution function to estimate the fast and slow closed-time constants (τ_closed1_ and τ_closed2_).

### Statistics

All data were tested for normality with the Shapiro -Wilk test and either parametric or non- parametric statistical tests were performed. For parametric tests, one-way ANOVA with Bonferroni’s post-hoc test was performed. For non-parametric tests, the Kruskal–Wallis test with Dunn’s multiple comparisons test were performed. Po-V curves were analyzed separately, where data at each potential were compared using multiple Mann–Whitney tests with corrections for multiple comparisons. Statistical significance was determined at *p* < 0.05 using Prism v10.0.0. Data are reported as group mean ± SEM.

## Results

### Case 1: undefined novo KCNMA1 N999S variant associated with epilepsy and dyskinesia

Case 1 (female) was aged 3 years, 7 months at the time of her evaluation at the NIH clinical center. She presented at 11 months with recurrent episodes of loss of motor tone in her neck and upper and occasionally lower extremities. She also had tracheomalacia, severe myopia, exotropia, bilateral ptosis, average verbal and non-verbal reasoning and low normal spatial reasoning and gross motor function, joint hypermobility, and frequent infections during the first year of life. She continued to experience 50 to hundreds of episodes per day of loss of tone that were considered frequent atonic seizures, imposing a significant impact on daily functioning. On admission, her physical examination revealed a child with acquired microcephaly, bilateral ptosis and severe myopia, facial asymmetry with right-sided weakness, ears with overfolded major helices, joint hypermobility with bilateral pes planus, and rash/ulcerations to her feet but no other cutaneous changes.

An awake electroencephalogram (EEG) and overnight sleep study with full EEG leads revealed generalized and multifocal discharges suggesting an increased risk for generalized or multifocal seizures, and mild disorganization of background, suggesting mild diffuse or multifocal cerebral dysfunction. A daytime polysomnogram, with leads placed at the chin, right and left legs to assess for loss of tone, captured seven typical events with brief altered awareness and apparent loss of axial tone. These events did not correlate with the frequent epileptiform discharges during her EEG indicating atonic seizures. The events did not reveal features of cataplexy. The patient was diagnosed clinically with paroxysmal nonkinesigenic dyskinesia with or without generalized epilepsy.

Whole-genome sequencing did not identify evidence for differential diagnoses of Fragile X, narcolepsy, or NPC2-related gene associations. However, the proband was heterozygous for a *de novo* missense variant in KCNMA1 on chromosome 10: g.78651467T > C (GRCh37); c.2996A > G; p.Asn999Ser (Table 1). Additional genetic findings were identified in NPC1, RNF31 variants, and TNXB. The *KCNMA1* variant was considered pathogenic for paroxysmal dyskinesia diagnosis (PNKD3; OMIM # 609446), an autosomal dominant disorder characterized by atonic falls or nodding and occasionally abnormal eye movements beginning in early childhood [21].

**Table 1.** Patient genotypes and clinical information.

Case	Variant	rsID	Allele Frequency
1	c.2996A > G; p.(Asn999Ser) (het)#	rs886039469	absent
2	c.1967A > C; p.(Glu656Ala) (het)*	rs149000684	0.000004389

Het: Heterozygous, *de novo;* # Reference Sequence: NM_001014797.2. Additional genetic findings include: c. *68 G > A in *NPC1* (NM_000271.5), two inherited *RNF31* variants (c.1865A > T, p.(Gln622Leu) and c.3106 G > T, p.(Val1036Leu); NM_017999.5), two inherited *TNXB* variants (c.113 G > A, p.(Arg38Gln) and c.8536 G > C, p.(Gly2846Arg); NM_019105.8).

*Reference Sequence: NM_002247.2. Additional genetic findings include: heterozygous c.1354 G > A, p.(Val452Met) in *GPC3* (NM_001164617.1), heterozygous c.5108C > G, p.(Pro1703Arg) in *RELN* (; NM_005045.3), heterozygous c.2275 G > A, p.Val759Leu in *VPS13B* (NM_017890.4), and homozygous c.3640del, p.(Cys1214Alafs *31) in *EXPH5* (NM_015065.2).

rsID: Reference SNP cluster ID from dbSNP (https://www.ncbi.nlm.nih.gov/snp/)

Allele frequencies were obtained from gnomAD v4.1 (https://gnomad.broadinstitute.org/).

### Case 2: undefined novo KCNMA1 E656A variant associated with epilepsy and dyskinesia

Case 2 (39-year-old female) is a 39-year-old adopted female with a complex medical history of failure to thrive, hyperextensible joints with pain, paroxysmal episodes of loss of consciousness, autism spectrum disorder, and fragile skin. Vitals were normal but she was using nasal cannula oxygen supplementation when assessed in person. She reports a history of infantile spasms, epilepsy from age 7 years old, and motor dysfunction (hypotonia, myoclonus, and dyskinesia). No EEG evaluation was present in the medical record. Developmental delays in motor, speech, writing, and independent activities and intellectual disability were reported. Dysautonomia, urinary (nocturia), gastrointestinal (diarrhea, nausea and vomiting), sensory (hypersensitivity to smells and sounds), and sleep abnormalities were reported. Respiratory symptoms reported include shortness of breath with mold, grass, pollen and other strong odors. MRI of the brain without contrast at age 18 was normal. No visceral structural abnormalities were reported except a high arched palate. Echocardiograms were performed after two separate events of chest tightness, sweating, and pain as an adult and reported as normal with no dilatation of the root of the aorta. Blistering, eczema, and dryness were reported in the skin, and she was diagnosed with Epidermolysis Bullosa and Ehlers–Danlos syndrome. A skin biopsy at age 12 showed vacuolization of basilar keratinocytes, clumps of keratin within cytoplasm, and few small areas of clefts along the dermal-epidermal junction. Type 4 collagen was present at the base of the cleft.

Behavioral issues started at age 10 with the threat of suicide followed by hospitalization and subsequent psychiatric therapy and medications. Menarche was recalled at age 14 years old. An increased frequency of headaches, mood swings, and seizures were reported during menses. A second psychiatric hospitalization occurred at age 15. She was weaned off psychiatric medication at age 16 and subsequently diagnosed with autism at age 18–19 years old. However, her family reports she has always had poor social interactions and emotional lability and continues to engage in both self-stimulatory and self-injurious behaviors (head banging).

The proband was heterozygous for a missense variant in KCNMA1, c.1967A > C; p.Glu656Ala (Table 1). Additional genetic findings were identified in GPC3, RELN, VPS13B, and EXPH5. The *KCNMA1* variant pathogenicity was considered inconclusive with respect to any of the diagnoses in Case 2. Only one other patient was reported with this variant, and this individual had severe epilepsy of multiple types at age 30 [12]. However, functional testing did not show differences in channel activity or properties from control, and the role of this variant in the epileptic phenotype was not clearly supported. Therefore, additional evidence is needed to assess the pathogenicity of E656A.

### BK channel expression from patient-derived KCNMA1^N999S/+^ and KCNMA1^E656A/+^ skin fibroblasts

The levels of mutant and wild-type *KCNMA1* transcripts in primary human tissue with heterozygous N999S and E656A genotypes were first assessed. RNA was isolated from *KCNMA1*^N999S/+^ and *KCNMA1*^E656A/+^ skin fibroblasts obtained from Cases 1 and 2, respectively. Two fibroblast lines with no reported *KCNMA1* variants from sex-matched unrelated individuals obtained from the Coriell repository, Control-1 (Ctrl-1; Coriell GM00038H) and Control-2 (Ctrl-2; Coriell GM01652F), were used as controls (Table 1). *KCNMA1*^N999S/+^ fibroblasts grew normally, but anecdotal observations suggested *KCNMA1*^E656A/+^ from Case 2 grew more slowly. Libraries were prepared from 3 to 7 ×10^6^ cells per sample and over 300 million reads per sample were generated. The reference positions corresponding to *KCNMA1* E656A and N999S had coverage of 230–1100 reads. *KCNMA1*^N999S/+^ fibroblasts expressed WT and N999S alleles in approximately equal ratios (Figure 1(B)). However, the E656A mutation was detected in only about a third of the reads from *KCNMA1*^E656A/+^ fibroblasts (Figure 1(C)), suggesting differences in transcript production or stability. The number of reads was also lower for E656A than for the other alleles. This suggests that the E656A mutation affects the number of mutant subunits available to form BK channels, while the N999S mutation does not.

### BK channel properties from patient-derived KCNMA1^N999S/+^ and KCNMA1^E656A/+^ fibroblasts

**Figure 2.**
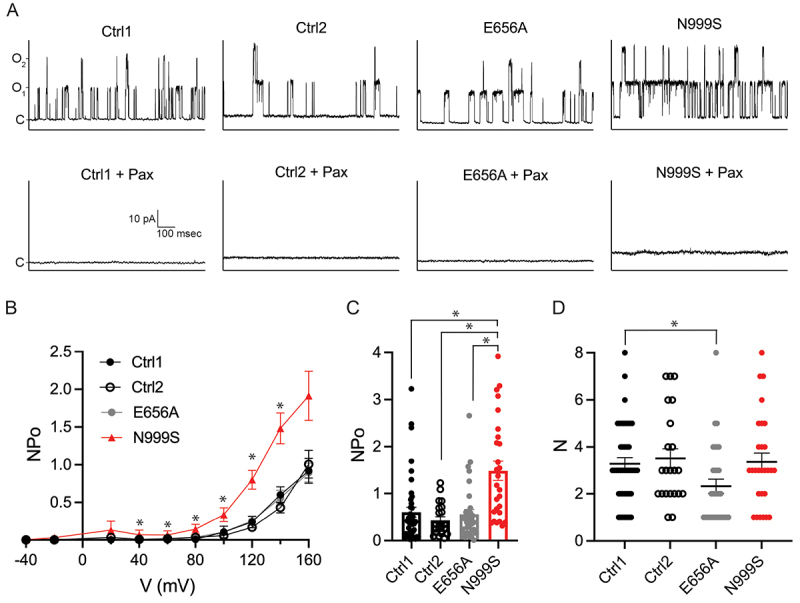
Comparison of BK channel properties from WT^GM00038H^, WT^GM01652F^, *KCNMA1*^E656A/+^, and *KCNMA1*^N999S/+^ fibroblasts. (A) top panels, representative traces of BK currents elicited at a membrane potential of 120 mV. NPo calculated from 15 s of recording. C, O_1_ and O_2_ indicate the current when channels are closed, a single channel is open, and two channels are open, respectively. Bottom panels, the same patches after addition of 100 nM paxilline. (B) NPo versus membrane potential for BK channels from WT^GM00038H^ (Ctrl-1, black closed circles, N = 43), WT^GM01652F^ (Ctrl-2, black open circles, N = 22), *KCNMA1*^E656A/+^ (E656A, grey circles, N = 27), and *KCNMA1*^N999S/+^ (N999S, red triangles, N = 32) fibroblasts. Data are mean ± SEM. From 40 to 140 mV, Ctrl-1 and Ctrl-2 NPos were significantly different from that of N999S, p < 0.05; from 60 to 160 mV, E656A NPos were significantly different from that of N999S, *p < 0.05; t-test with *p* values adjusted for multiple comparisons. (C) NPo at 140 mV for BK channels from the indicated fibroblast lines. Ctrl-1 versus N999S, *p < 0.0001; Ctrl-2 versus N999S, *p = 0.0002; E656A versus N999S, *p = 0.0003, Kruskal-Wallis with Dunn’s multiple comparison. Data are individual values with mean ± SEM. (D) number of channels per patch. Ctrl-1 versus N999S, *p = 0.0481, Kruskal-Wallis with Dunn’s multiple comparison. Data are individual values with mean ± SEM.

To determine if the consequences of the N999S and E656A *KCNMA1* mutations can be detected from endogenous BK channels in human skin fibroblasts, electrophysiology was used to assess channel activity. BK currents from control, *KCNMA1*^N999S/+^ and *KCNMA1*^E656A/+^ fibroblasts were assessed using cell-attached patch-clamp (Figure 2). BK channel openings were recorded for 15 s over a range of membrane potentials (−40 mV to +160 mV) from each cell. The external bath solution contained a high K^+^ concentration to maintain the cytoplasmic potential close to 0 mV and to distinguish channel openings. Multiple channel openings were observed (Figure 2(A), top panels) that activated in a voltage-dependent manner (Figure 2(B)). These openings were abolished with the BK channel inhibitor paxilline (100 nM, Figure 2(A), bottom panels): Ctrl-1 (*N* = 29), Ctrl-2 (*N* = 5), *KCNMA1*^E656A/+^ (*N* = 7), and *KCNMA1*^N999S/+^ (*N* = 12) fibroblasts.

The ensemble BK channel open probability (NPo) was estimated from the number of channels in each trace (N) multiplied by the single channel open probability (Po), where Po is calculated from the total time the channel is open (O) and closed (C): O/(O+C). The NPo-voltage relationship for these patches is shown in Figure 2(B). In these cell-attached recording conditions, BK channel currents from control fibroblasts were activated at voltages greater than +80 mV, under endogenous cytosolic Ca^2+^ (previously estimated around ~100 nanomolar in this cell type [22–24]). Ctrl-1 and Ctrl-2 fibroblasts showed no significant differences in NPo from each other, or from *KCNMA1*^E656A/+^ fibroblasts at any voltage (Figure 2(B,C)). In contrast, the NPo for channels from *KCNMA1*^N999S/+^ fibroblasts were larger than those from Ctrl-1, Ctrl-2, and *KCNMA1*^E656A/+^ fibroblasts at voltages equal to or higher than +40 mV and were significantly larger than those from *KCNMA1*^E656A/+^ fibroblasts starting at +60 mV. At +140 mV, channel NPos from *KCNMA1*^N999S/+^ fibroblasts were 2.5–3.4 times as large as those from Ctrl-1, Ctrl-2, or *KCNMA1*^E656A/+^ fibroblasts (Figure 2(C)). These data indicate that channels from *KCNMA1*^N999S/+^ fibroblasts showed a GOF phenotype in the ensemble activity recorded from multi-channel patches, while channels formed in *KCNMA1*^E656A/+^ fibroblasts did not.

The increased NPo from channels in *KCNMA1*^N999S/+^ fibroblasts could result from a change in the number of BK channels (N) or an increase in the single channel open probability (Po). To assess these possibilities, the number of channels per patch was determined (Figure 2(D)). Ctrl-1, Ctrl-2, and *KCNMA1*^N999S/+^ fibroblasts had similar numbers of channels per patch, with an average of 3.2, 3.5, and 3.4 channels per patch, respectively. In contrast, the number of channels was lower for *KCNMA1*^E656A/+^ fibroblasts (an average of 2.3 channels per patch). No decrease in the number of channels per patch was previously reported in prior studies of homomeric channels containing the E656A mutation expressed in heterologous cells [12,15]. This result, along with the RNA-Seq results shown in Figure 1, suggests that the E656A mutation affects BK channel expression in human fibroblasts, whereas the N999S mutation does not.

### KCNMA1^N999S/+^ single channel properties

**Figure 3.**
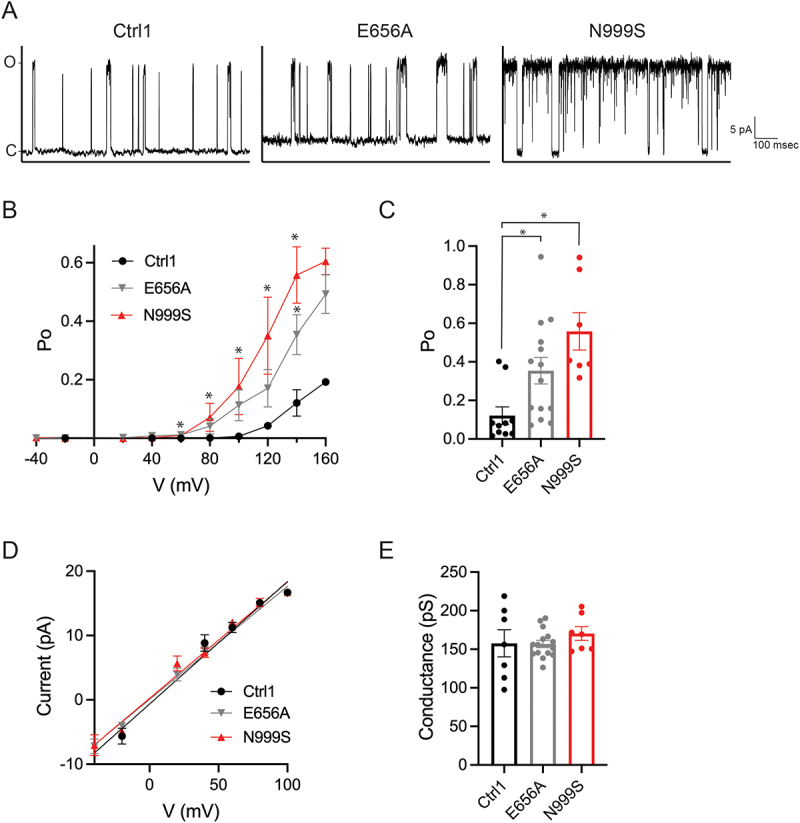
Comparison of single channel opening and conductance properties of BK channels from WT+^GM00038H^, *KCNMA1*^E656A/+^, and *KCNMA1*^N999S/+^ fibroblasts. (A) representative single-channel traces. Traces elicited at V_pip_ = 120 mV. (B) Po versus membrane potential for single BK channels from WT^GM00038H^ (Ctrl-1, black closed circles, N = 10), *KCNMA1*^E656A/+^ (E656A, grey circles, N = 14), and *KCNMA1*^N999S/+^ (N999S, red triangles, N = 7) fibroblasts. From 60–140 mV, ctr-1 Po was significantly different from that of N999S, *p < 0.01; at 140 mV, Ctrl-1 Po was significantly different from that of E656A, *p < 0.05; t-test with *p* values adjusted for multiple comparisons. Data are mean ± SEM. (C) NPo at 140 mV for BK channels from the indicated fibroblast lines. Ctrl-1 versus E656A, *p = 0.0184; Ctrl-1 versus N999S, *p = 0.002; Kruskal-Wallis with Dunn’s multiple comparison. Data are individual values with mean ± SEM. (D) single-channel current versus voltage for BK channels from Ctrl-1 (N = 7), E656A (N = 14), and N999S (N = 7) fibroblasts. Data are mean ± SEM. (E) single-channel conductance for BK channels from the indicated fibroblast lines calculated from the slope of plots in (D). p > 0.05, ANOVA with Tukey’s multiple comparison. Data are individual values with mean ± SEM.

Next, we analyzed recordings from patches containing only one BK channel (representative traces are shown in Figure 3A). When our study was limited to single-channel recordings, BK channels from *KCNMA1*^N999S/+^ fibroblasts still showed a significant increase in Po compared to Ctrl-1 at multiple voltages (Figure 3(B,C)). There were too few patches with single channels from Ctrl-2 to include in our analysis. This increase was further consistent with estimates of the single-channel Po derived from multi-channel patches, where NPo was divided by N (Supplemental Figure 1(A,B).

**Figure 4.**
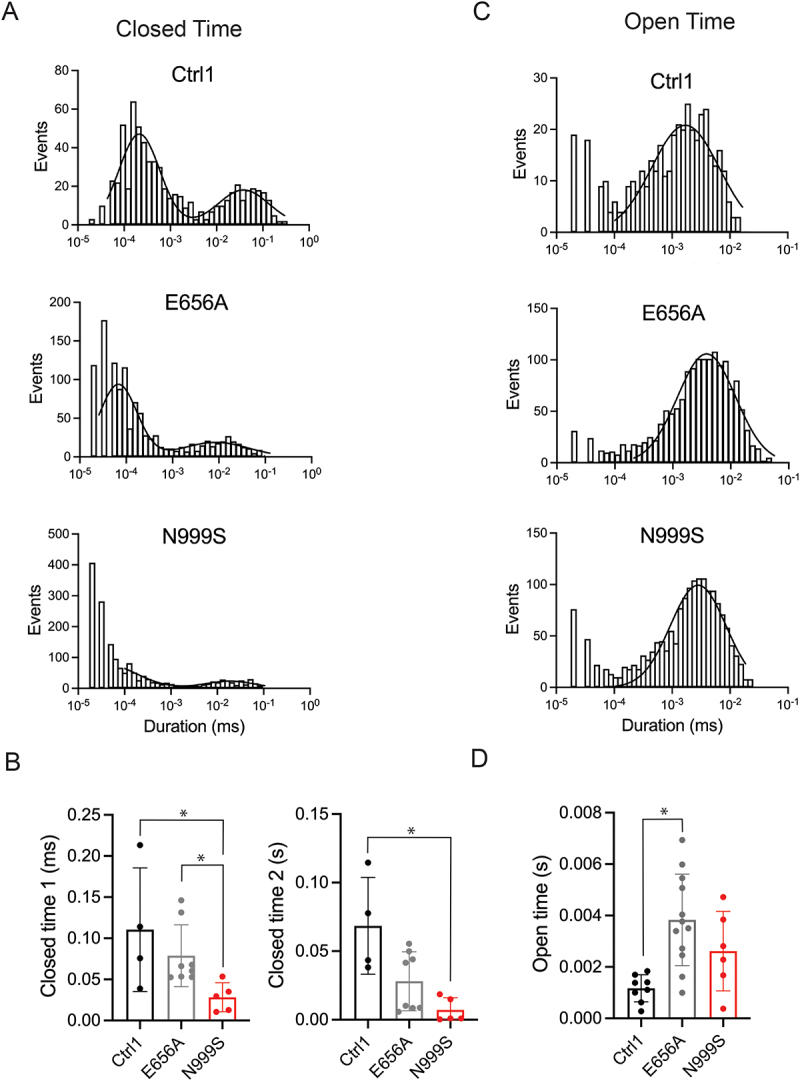
Single channel open and closed time analysis. (A) representative closed time distributions from Ctrl-1 (top), *KCNMA1*^N999S/+^ (middle) and *KCNMA1*^E656A/+^ (bottom) recordings. (B) fast (τ_closed1_, left) and slow (τ_closed2_, right) closed-time constants for BK channels from the indicated fibroblast lines calculated from fitting a two-component (double) log-normal distribution function. τ_closed1_: ctrl-1 versus N999S, *p = 0.0320; N999S versus E656A, *p = 0.0390, Kruskal-Wallis with Dunn’s multiple comparison. τ_closed2_: ctrl-1 versus N999S, *p* = 0.0207, Kruskal-Wallis with Dunn’s multiple comparison. Data are individual values with mean ± SEM. (C) representative open time distributions. (D) open-time constants (τ_open_) calculated from fitting using a single-component log-normal distribution function. Ctrl-1 versus E656A, *p = 0.0016, ANOVA with Tukey’s multiple comparison. Data are individual values with mean ± SEM.

The increased Po suggested that BK channels from *KCNMA1*^N999S/+^ fibroblasts spend more time in the open state. Previous studies also showed that homomeric N999S-containing channels in heterologous systems showed an increase in the open dwell time [12]. To investigate this factor in the *KCNMA1*^N999S/+^ fibroblasts, dwell time histograms were constructed from single-channel patches and fitted with curves to obtain open and closed time constants. Representative histograms are shown in Figure 4(A,C). BK channels from *KCNMA1*^N999S/+^ fibroblasts exhibited a significantly shorter closed time constant compared to those from Ctrl-1 fibroblasts (0.019 ± 0.038 ms versus 0.049 ± 0.019 ms for closed time 1 and 0.043 ± 0.064 ms versus 0.0091 ± 0.013 s for closed time 2, respectively) (Figure 4(B)). Mean open time constant increased for BK channels from *KCNMA1*^N999S/+^ when compared to Ctrl-1 fibroblasts; however, the difference was not statistically significant (Figure 4(D)). These data show the changes in closed and open time constants associated with the increased Po in *KCNMA1*^N999S/+^ fibroblasts, corroborating studies with N999S-containing channels expressed in heterologous cells [12].

We next determined whether BK single-channel conductance was altered by calculating the slope conductance for single-channel currents from −40 mV to +100 mV. The single-channel conductance for Ctrl-1, Ctrl-2, and *KCNMA1*^N999S/+^ derived from single (Figure 3(D,E)) and multi-channel patches (Supplementary Figure S1D, E) were indistinguishable (Ctrl-1, 162 ± 4 pS, *N* = 47; Ctrl-2, 167 ± 6 pS, *N* = 21; *KCNMA1*^N999S/+^, 167 ± 6 pS, *N* = 29). This lack of change in single-channel conductance was consistent with previous studies in homomeric heterologously expressed channels [12,15]. These data demonstrate that BK channels from heterozygous *KCNMA1*^N999S/+^ fibroblasts exhibit increased single channel open probabilities and decreased closed times, with no change in the number of channels recorded (Figure 2(D)) or single-channel conductance (Figures 3(D,E) and Supplementary Figure S1D, E).

### KCNMA1^E656A/+^ single channel properties

Analysis of single channels from *KCNMA1*^E656A/+^ fibroblasts also revealed an increase in Po compared to Ctrl-1 (Figure 3(A–C), indicating a GOF effect of the E656A mutation on channel activity. Histogram analysis did not show a significant decrease in closed time constants for channels from *KCNMA1*^E656A/+^ fibroblasts as compared to those from Ctrl-1 fibroblasts (Figure 4(B)), but did show a large and significant increase in open time constants (Figure 4(D)). The increase in single-channel Po from *KCNMA1*^E656A/+^ fibroblasts was also corroborated in recordings from multi-channel patches (at 160 mV), in comparison to both Ctrl-1 and Ctrl-2 (Supplemental Figure 1(A,C)). However, this observation contrasts with the overall NPo originally observed in multi-channel patches from *KCNMA1*^E656A/+^ fibroblasts, which was not distinguishable from the control patches (Figure 2(B,C)). The discrepancy between single and multi-channel patch Pos suggests that the decreased number of channels per patch (Figure 2(D)) may have masked the observation of a GOF change in NPo from *KCNMA1*^E656A/+^ channels.

Lastly, the single-channel conductance assessed from the slope conductance of single (Figure 3(D,E)) or multi-channel currents (Supplementary Figure S1D, E) from *KCNMA1*^E656A/+^ fibroblasts (164 ± 3 pS, *N* = 32) was not different from Ctrl-1, Ctrl-2, or *KCNMA1*^N999S/+^. These data demonstrate that BK channels from heterozygous *KCNMA1*^E656A/+^ fibroblasts demonstrate increased single channel open probabilities and increased open time constants, a decrease in the number of channels recorded, and no change in the single-channel conductance.

## Discussion

Identifying and characterizing patient-associated variants is critical for understanding the monogenic basis of *KCNMA1*-linked channelopathy. Here, we report the detection of single BK channel currents from the primary skin fibroblasts patients presenting with epilepsy and dyskinesia. BK channels were detected consistently in both unaffected controls and in fibroblasts obtained from two affected individuals harboring either the N999S or E656A *KCNMA1* variants. Additionally, each respective mutation was detectable in transcripts prepared from the fibroblasts. These results demonstrate that fibroblasts obtained from skin punches can be used to evaluate the associated alterations in endogenous BK channel expression and single-channel activity. The data in this study support pathogenicity for both variants in the affected heterozygous individuals. Only one prior study has investigated the impact of a *KCNMA1* mutation in cells obtained from an affected individual. This individual harbored a *de novo* balanced chromosomal translocation causing *KCNMA1* haploinsufficiency, and macroscopic BK currents were assessed from a patient-derived lymphoblastoid cell line [25]. However, the study did not distinguish functional alterations at the single BK channel level. Thus, it is unknown whether the observed reduction in BK currents from patient cells was the result of a reduced number of functionally expressed BK channels, changes in channel composition and gating, or other pathological changes associated with chromosomal rearrangement [26].

The N999S variant was selected for study based on the availability of patient tissue, its higher prevalence compared to other disease-associated *KCNMA1* mutations, and the consistently strong GOF channel properties in heterologous cells [5,6,12,15,16,27] and in heterozygous *KCNMA1*^N999S/+^ mice [5]. Thus, we hypothesized that strong GOF channel activity was likely to be detectable in *KCNMA1*^N999S/+^ skin fibroblasts, serving as a positive control. The results in this study demonstrate that heterozygous *KCNMA1*^N999S/+^ fibroblasts produce BK channels with GOF activity at the single channel level and corroborate that this GOF activity results from gating effects (increased Po and decreased closed time). Changes in transcript levels, number of channels, or single-channel conductance did not play a significant role in the GOF phenotype in *KCNMA1*^N999S/+^ patient-derived cells.

In contrast to N999S, the pathogenicity for many patient-associated *KCNMA1* variants is not known, as was the case with E656A. Additional genetic findings and inconsistent data from expression of homomeric mutant channels in heterologous cells [12,15] made understanding the functional consequences of this mutation difficult. Investigation in patient-derived fibroblasts helped resolve these complexities and support the classification of this variant as likely pathogenic (LP). Surprisingly, heterozygous *KCNMA1*^E656A/+^ fibroblasts produced BK channels with GOF activity resulting from increased gating. While these channels have increased Po and increased open time, corroborating previous a previous study [15], the GOF phenotype in multi-channel patches was mitigated by a concurrent decrease in the number of channels recorded. A reduced number of functional channels in *KCNMA1*^E656A/+^ fibroblasts was consistent with the reduced E656A transcripts levels compared to the two other alleles tested. It is possible that decreased expression could mitigate some of the GOF effects in patient tissues. Another *KCNMA1* variant has been shown to exhibit changes in BK channel properties with the potential offset each other; the G375R variant is considered GOF with respect to voltage activation and LOF in single-channel conductance [18]. Although the relative expression of G375R and wild-type alleles has not been studied in patient tissues, like E656A, its penetrance would depend on the integration of these phenotypes and subunit stoichiometry in specific neurons.

N999S produces GOF macroscopic BK currents, changes in neuronal firing, and neurobehavioral pathology in a heterozygous transgenic mouse model, and the results from single channels recorded in this study suggest that heteromeric BK channels are present in heterozygous patient tissues. Direct sequencing of transcripts revealed a 1:1 expression ratio for WT and N999S alleles in *KCNMA1*^N999S/+^ fibroblasts. Coupled with single-channel recordings validating the GOF properties, it can be inferred that most fibroblast *KCNMA1*^N999S/+^ channels contain at least one mutant subunit. Consistent with this idea, the shift in the Po-V curve of BK channels in *KCNMA1*^N999S/+^ fibroblasts is less than for single homomeric BK^N999S^ channels recorded in heterologous cells [12]. However, because the recording conditions differ and the splice isoform and subunit stoichiometry are not known in either study, direct comparisons are difficult. Nevertheless, intermediate phenotypes have been associated with WT:mutant heterotetramers in a previous study, where transcripts containing the *KCNMA1*-G375R patient variant were expressed at equal ratios with the WT transcript in *Xenopus laevis* oocytes and five different stoichiometries producing GOF channel properties were observed [18]. Understanding how WT and mutant BK channel subunits assemble in the neuronal context, and any implications for pathogenicity, will require future studies.

Accurate genotype-phenotype correlation in *KCNMA1* neurological disease, like other rare disorders, is limited by lack of access to patient neurons. Future studies employing cellular reprogramming could facilitate the study of BK channels in neurons derived from skin fibroblasts [28]. This would further facilitate the identification of BK channel properties relevant in neurological disease, since fibroblasts exhibit differences in isoform expression and biophysical properties from neurons. This study provides proof-of-principle that functional characterization of *KCNMA1* variants in endogenous BK channels in the context of patient zygosity is possible from skin fibroblasts, supporting more robust genotype-phenotype correlations. Such investigations in patient tissues may also better address the phenotypic variability in K*CNMA1* channelopathy that can arise from differences in genetic backgrounds between patients [29–32].

## Supplementary Material

SupplementalFigure1.tif

## Data Availability

Deidentified data and materials from this study will be made available upon reasonable request.
