# Supplementary figures and images for: BK channel activity in skin fibroblasts from patients with neurological disorder
Source: Channels (Austin). 2025 Aug 10;19(1):2542811. doi: 10.1080/19336950.2025.2542811 (PMC12341059; doi:10.1080/19336950.2025.2542811)

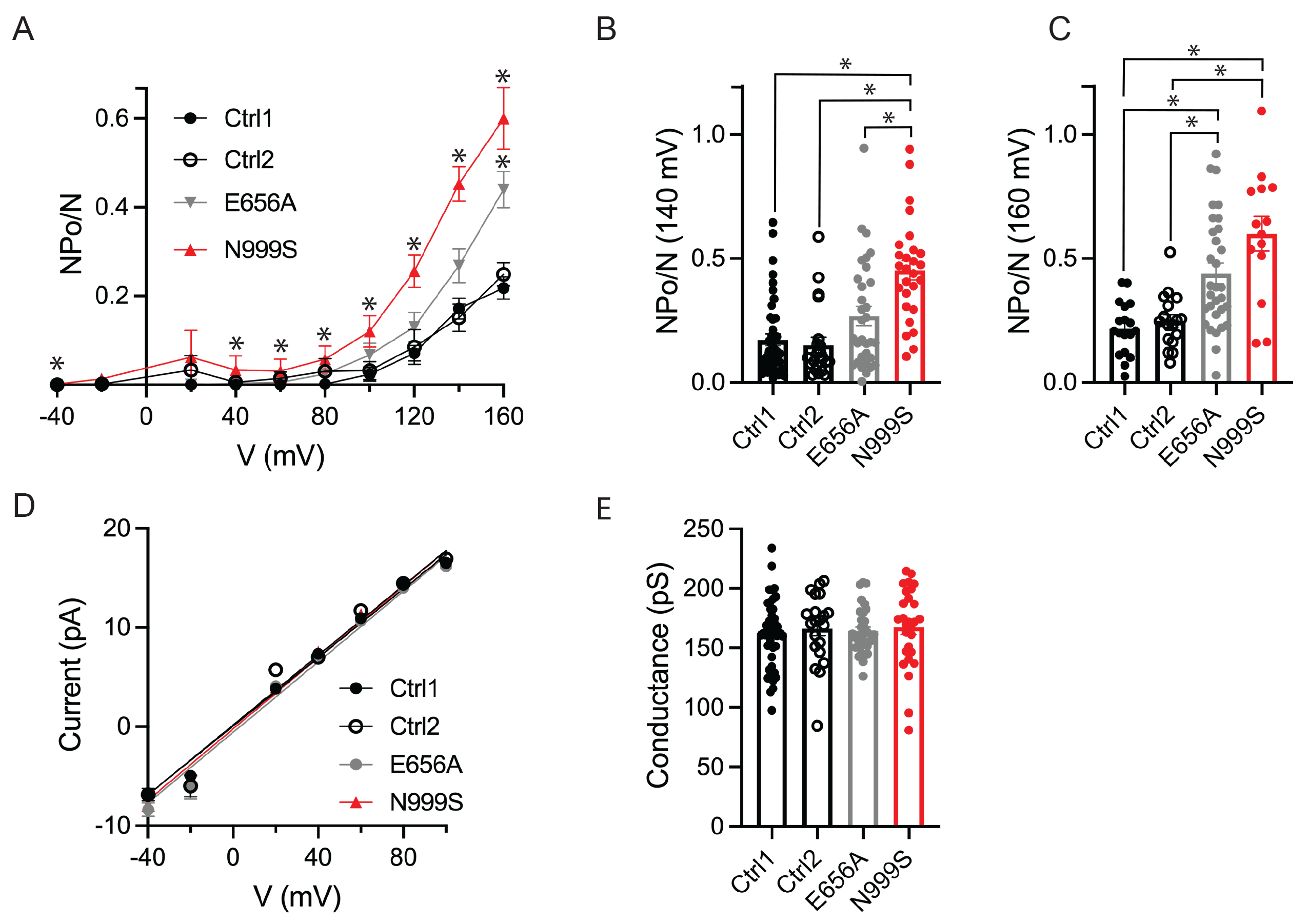

Supplement: SupplementalFigure1.tif [file KCHL_A_2542811_SM8077.tif]
